# Supplementary material for: Accessibility to Occupational Therapy Services for Hereditary Transthyretin Amyloidosis
Source: Int J Environ Res Public Health. 2022 Apr 7;19(8):4464. doi: 10.3390/ijerph19084464 (PMC9031487; doi:10.3390/ijerph19084464)
Supplement: Supplementary file 1 [file ijerph-19-04464-s001.zip › ijerph-1604083-supplementary.pdf]

OCCUPATIONAL THERAPY

The objective of this questionnaire is to develop an investigation related to the perception of patients on the benefits of Occupational Therapy and diagnosis of Hereditary Transthyretin Amyloidosis.

1. I agree to participate in the study

- Yes
- No

2. Age

____

3. Gender

- Female
- Male
- Prefer not to say

4. Age of disease debut

____

5. Age at diagnosis

____

6. Disease stage

- ( ) 0 I No impairment
- ( ) I Sensory symptoms, walking normally
- ( ) II Walking difficulties, does not require support or cane
- ( ) IIIA one stick or crutch needed for ambulation
- ( ) IIIB Two sticks or two crutches needed for ambulationIV
- ( ) patient confined to a bed or wheelchair
- ( ) Other: ______________________________

7. Which are your main symptoms?

Mark all that apply.

- [ ] Pain or loss of sensation in feet / legs
- [ ] Foot sores
- [ ] Weakness in legs
- [ ] Impotence (erectile dysfunction, in men)
- [ ] Difficulty with fine motor skills (buttoning a shirt)
- [ ] Depression
- [ ] Loss of cold/heat sensation
- [ ] Constipation or diarrhea
- [ ] Lack of appetite or anorexia
- [ ] Weightloss
- [ ] Abnormal sweating
- [ ] Difficulty in vision
- [ ] Dizziness or feeling dizzy
- [ ] Decreased tolerance to exercise
- [ ] Breathing difficulty when lying down
- [ ] Tiredness or malaise
- [ ] Heart Palpitations
- [ ] Anxiety
- [ ] Bladder infections
- [ ] Nausea/vomiting

Other (describe): [ ]

In relation to your occupational abilities

8. Indicate the level of difficulty in performing the Basic Activities of Daily Living (grooming, dressing, eating, functional mobility to perform basic tasks)

		2	3	4	5	
No difficulty	○	○	○	○	○	Much difficulty

9. Indicate the level of difficulty to perform the Instrumental Activities of dailylife (cooking, washing clothes, handling medication, shopping, using public transport)

		2	3	4	5	
No difficulty	◯	◯	◯	◯	◯	Much difficulty

10. Indicate the level of difficulty to carry out Advanced Activities of daily life (work, travel, active social participation)

		2	3	4	5	
No difficulty	◯	◯	◯	◯	◯	Much difficulty

11. Briefly indicate your current significant occupations (work, leisure and free time)

____

12. What do you think about the possible benefits of Occupational Therapy?

13. ¿Do you know what the Occupational Therapy professional does?

- ◯ Yes
- ◯ No

14. ¿Have you used the services of an Occupational Therapist?

- Yes
- No

15. If you have used the services of an Occupational Therapist, ¿when did you start using them?

- Before the diagnosis of your illnessAt
- the diagnosis of your illness
- Two years after the diagnosis of your illness
- More than two years after the diagnosis of your illnessOthers
-

16. 16. If you have used the services of an Occupational Therapist, what type of interventions have been applied?

- Program of physical exercise and mobilizations to improve strength, motor skills, posture and balance
- Education program to adapt your tasks and simplify them
- Education program to conserve your energy and be able to carry out your dailytasks
- Recommendations to adapt your home and facilitate your daily life and use of assistive products
- Occupational motivation program
- Other (describe): ____________________

17. If you have used the services of an Occupational Therapist, how many years have you used the services of an Occupational Therapist?

- I am currently using them
- I have used them in the last year
- I used them more than a year ago
- I used them more than three years agoI
- used them more than five years ago
- Otro: ________________

If you have never used the services of an Occupational Therapist and knowing that an Occupational Therapist is in charge of, promoting the maximum independence and quality of life of the person through occupation. Please indicate from 1 to 5 whether or not you agree with the following statements.

18. Do you consider that the inclusion of an Occupational Therapist as part ofthe multidisciplinary team could be beneficial for the management of his pathology

		2	3	4	5	
Totally disagree	○	○	○	○	○	Totally agree

19. If you have already used the services of an Occupational Therapist:

20. Do you consider that having turned to an Occupational Therapist was beneficial for you?

		2	3	4	5	
Totally disagree	○	○	○	○	○	Totally agree
